# Reducing the Use of Disposable Plastics through Public Engagement Campaigns: An Experimental Study of the Effectiveness of Message Appeals, Modalities, and Sources

**DOI:** 10.3390/ijerph19148273

**Published:** 2022-07-06

**Authors:** Marko M. Skoric, Nan Zhang, Juma Kasadha, Chun Hong Tse, Jing Liu

**Affiliations:** 1 Department of Media and Communication, City University of Hong Kong, Hong Kong 999077, China; mskoric@cityu.edu.hk (M.M.S.); jkasadha2-c@my.cityu.edu.hk (J.K.); 2 School of Journalism and Communication, Xiamen University, Xiamen 361005, China; 3 School of Journalism and Communication, The Chinese University of Hong Kong, Hong Kong 999077, China; chtse26-c@my.cityu.edu.hk; 4 Department of Economics, Hong Kong Baptist University, Hong Kong 999077, China; jingliu@hkbu.edu.hk

**Keywords:** single-use plastics, disposable plastics, message framing, experiment

## Abstract

This study examines how different ways of presenting information about the ecological threats stemming from the use of disposable plastics may affect people’s willingness to reduce their use. To test our hypotheses, we used a 2 × 3 × 2 between-subjects experimental design, utilizing a sample of 1001 Hong Kong residents. The independent variables tested included: (a) message frame (gain vs. loss), (b) modality (text vs. image vs. infographic), and (c) information source (government vs. non-governmental organization). The findings demonstrate that the loss frame was more effective than the gain frame in persuading participants to reduce the use of disposable plastics. Furthermore, compared to image-based messages, text-based and infographic-based messages were more effective in promoting the reduction in the use of disposable plastics. For information sources, however, we found no main effect on behavioral intentions. However, this study still suggests an interaction effect of the loss frame and NGO source, as well as the interaction between text-based modality and government source, both leading to more positive outcomes. Furthermore, the study reveals that negative emotional responses mediate the effect of media frames on behavioral intentions. The findings offer useful insights for designing more effective communication campaigns aimed at curbing the use of disposable plastics.

## 1. Introduction

Single-use plastic waste poses a serious threat to the natural environment. Globally, it is estimated that over 380 million tons of plastics are produced every year, and up to 50% of that is for single-use purposes. Data show that more than 10 million tons of plastic are dumped into oceans annually, and more than 1 million marine animals are killed by it every year [1]. It is believed that about USD 2.5 trillion is lost because of marine plastic pollution each year [2]. In Hong Kong, plastic pollution is getting out of control, such that the city’s beaches and waterways are drowning in plastic, resulting in nearly 6000 marine species being considered endangered [3]. While there is little doubt that government intervention is needed to curb the use of disposable plastics, another important locus of change should be citizens’ attitudes, habits, and behaviors. Therefore, public engagement campaigns should try to mobilize various segments of the population to (a) recognize the nature and severity of the problem and (b) change specific behaviors to reduce the use of disposable plastics. In fact, public engagement campaigns are increasingly being adopted internationally by a range of governmental and non-governmental (NGO) organizations, including the European Commission and the World Wildlife Fund (WWF), with a goal of reducing the use of disposable plastics. Media messages were found to play an important role in educating the public and influencing citizens’ pro-environmental behaviors, with some countries actively using various digital media channels to promote green programs (e.g., the Plastic Watch online program initiated by the BBC). Given the gravity of the problem and the potential for mitigation via persuasive messages, the key goal of this study was to provide empirically derived strategies for effective public engagement campaigns aimed at reducing plastic pollution. More specifically, this study aimed to identify the message characteristics that could help promote a reduction in the use of disposable plastics and encourage environmentally-friendly behaviors such as re-use and recycling among Hong Kong residents. Drawing on persuasion research, we focused on the three most frequently used message-related factors: (a) message frame (gain vs. loss), (b) modality (text vs. image vs. infographic), and (c) information source (government vs. non-governmental organization) [4,5,6].

Drawing on prospect theory [7], message framing is a persuasive communication strategy aimed at motivating behavior by emphasizing the benefits of engaging in the behavior (i.e., the gain frame) or the costs of not engaging in the behavior (i.e., the loss frame). Recent evidence suggests that people make decisions based on whether the behavior is perceived to involve risk [8,9,10]. Thus, people tend to embrace risk to minimize loss, while they are risk-averse when facing gain. Reducing the use of disposable plastics, likewise, can be framed by emphasizing either the benefits of it or the cost of not doing so. Although the bulk of previous studies used written text to present information, in recent years, we have seen the increasing use of visuals such as images and infographics in science and environmental communication [11,12,13]. According to this research, images and infographics play a pivotal role in persuasion because people’s risk perceptions of environmental hazards are strongly influenced by imagery [14]. As such, by considering the multimodal nature of digital media environments, we adapted a framework from the cue summation theory [15] and the dual-coding theory [16] to examine the persuasive effects of multichannel communications. This will help us understand whether the use of text versus images versus infographics strengthens or weakens persuasive outcomes when coupled with the use of different message frames. In addition, as digital technologies have lowered the cost and threshold of producing and disseminating information, messages are increasingly created and delivered by a wide range of sources. One of the consequences of this evolution is the increasing ambiguity surrounding the source and even the emergence of fake news [17]. People are likely to rely on source credibility to heuristically evaluate the information before making further decisions, especially when they lack knowledge of environmental issues [6,18]. This study thus also took into account the sender-related factors, with the aim to bridge source credibility research with the message strategy studies to generate insights into designing messages aimed at different stakeholders.

Lastly, we also examined the potential mediating role of emotional arousal (positive vs. negative), which is one of the key determinants of pro-environmental behaviors [19]. Although the influence of emotions has been overlooked within environmental research for a long time, recent studies have identified affective motives as important factors in affecting sustainable behaviors [20,21]. For instance, the literature indicates that emotions exert a crucial impact on rational processes [22] and may motivate environmental protection even more than data-intensive arguments [23]. In this study, we further examined how the mediated effects of positive and negative emotions affect citizens’ psychological well-being to be able to engage in environmentally sustainable behaviors that foster a reduction in single-use plastics.

## 2. Literature Review

Previous research has examined the drivers of environmentally sustainable behaviors in various social and cultural contexts. For instance, Heidbreder, Steinhorst, and Schmitt conducted an experimental study to investigate whether the “Plastic Free July” campaign could reduce plastic consumption in German, with the result that participants exposed to information about this campaign were significantly less likely to use disposable plastics than the control group who did not receive this information [24]. Walker and his colleagues’ study employed a survey in Canada, finding that Canadians were less willing to pay for sustainable food packaging alternatives but were highly motivated to reduce single-use plastic waste. They further discussed the relevant factors such as age, income, education, and region [25]. Through a case study of the campaign advocating banning single-use plastic straws in Brazil and the United States, Viera et al. suggested the best chance to drive a positive environmental change is to educate people on green consumption and how to discard waste properly [26]. These studies suggest the importance of conducting environmental campaigns to improve citizens’ awareness of the importance of environmental protection.

### 2.1. Gain- vs. Loss-Frame Messaging

Goal framing is a type of framing effect that is frequently employed in environmental communication studies addressing environmental sustainability [8,9,10,27,28]. By presenting information about the consequences of an action, goal framing emphasizes either the benefits (gains) associated with environmentally sustainable behaviors or the costs (losses) associated with unsustainable behaviors [29]. In their prospect theory, Tversky and Kahneman demonstrated that when facing a gain, people are risk-averse and prefer certainty; conversely, when facing loss, people embrace risk in an attempt to minimize loss [7]. In more detail, gain frames focus on the gains when a recommended behavior is performed, which may be presented as either obtained positive outcome or avoided negative outcome. In contrast, loss frames describe the losses of not performing that recommended behavior, which may be presented as either obtained negative outcome or avoided positive outcome [8]. The essential difference between the gain vs. loss frame lies in the positive or negative wording of the consequences ([30], p. 298). For instance, a possible gain frame in this study is, “If we reduce the consumption of single-use plastics, our living environment will be better and healthier.” The consequence—a better and healthier living environment—is positive. Conversely, a loss frame is to describe an undesirable and negative outcome, for instance, “If we do not reduce the consumption of single-use plastics, our living environment will become highly polluted and our health will be threatened.” Therefore, the gain frames stress that environmentally sustainable behaviors lead to local or overall benefits such as the improvement of the current environment. In contrast, loss frames indicate the threat by emphasizing the deteriorating situation caused by single-use plastics consumption [4].

Previous studies demonstrated the effectiveness of gain- and loss-frame messages in changing people’s behaviors. For instance, Cho and Bolster demonstrated the persuasiveness of loss-frame messages among smokers [31], and Mitkidis et al. demonstrated the effectiveness of loss framing on household pharmaceutical take-back schemes [32]. By affecting citizens’ cognitive processes, the loss frames with more extreme outcomes required more cognitive processing resources and were better remembered [33]. As for the gain-frame message, it was often evaluated as being more convincing when the message advocated a relatively less risky and simple action [31], such as exercising regularly [34,35], using sunscreen [36], and eating plant-based meals [37]. However, some studies found no statistically significant difference between messages with the gain vs. loss frames [30,38]. Due to the mixed pattern of findings from the extant research, we proposed testing the following research question:

**RQ1:**  *Which frame (gain vs. loss) is more effective in promoting the reduction in the use of disposable plastics?*

### 2.2. Multimodality and Information Source

Previous studies demonstrated that multimodality is an important factor affecting information processing. According to the cue summation theory [15], multichannel communications provide additional cues for learning that may improve information acquisition. Similarly, the dual-coding theory proposes that different types of stimuli may be processed by distinct cognitive subsystems [16]. According to the theory, text and graphics are processed separately by independent subsystems, and the combination is likely to promote the recall of information when compared to either mode alone [39]. Empirical studies also demonstrated the benefit of multimodal presentation and provided evidence for the cue summation theory and the dual-coding theory. For instance, Griffin and Stevenson found that undergraduates recalled statistical information better when the new story was presented in both text and graphics than in either mode alone [40]. Similarly, Michas and Berry demonstrated that adult learners performed better when the learning materials were presented in both text and line drawings than in either modality alone [41]. The benefit of the multimodal presentation was also observed in children solving geometric puzzles [42] as well as in other demographic groups with different tasks [43,44]. While visually communicated messages help the audience interpret media content and engage in an extensive learning process and comprehension [45], the use of infographics promotes an easier understanding of the environmental information [12,45]. Based on the above, we proposed the following hypothesis:

**H1.**  *Media modality (i.e., text vs. image vs. infographic) has an impact on the reduction in the use of disposable plastics. More specifically, infographics will be more effective than (a) text- and (b) image-based messages in promoting the reduction in the use of disposable plastics.*

In addition, previous studies also demonstrated that the sender-related factors might influence information processing, especially when the audience lacks the motivation or knowledge to scrutinize messages about environmental issues [5,6]. Carl Hovland’s seminal work, for instance, showed that trustworthy sources could increase the learning of the message and thus affect persuasion [46]. Later theories of persuasion, such as the Elaboration Likelihood Model (ELM) [47] and the Heuristic-Systematic Model (HSM) [48], also demonstrated that audiences tend to rely on the sender’s credibility as important heuristic cues for decision making. Both the ELM and the HSM are major dual-process theories explaining how individuals evaluate information quality and credibility. In detail, the ELM describes attitude change as a consequence of either a high or low level of elaboration (i.e., level of cognition) via central or peripheral processing routes. HSM, in contrast, proposes a co-occurrence of two modes (heuristic and systematic) when people are processing information. Central or systematic processing involves effortful thinking, and argument quality plays a major role in persuasion, while peripheral or heuristic processing is less effortful, and the source’s expertise is more relevant in changing attitudes [13,19,49]. According to these two theories, expert sources produce more persuasion than inexpert sources when motivation and ability to think are relatively low [50]. For example, Petty et al. found that under low engagement conditions, undergraduate students were more likely to accept advocacy presented by a professor than by a student, which illustrates the cue role for sources [50]. Additionally, Priester and Petty pointed out that message sources can induce a general sense of certainty or doubt, and people think more about messages from sources they feel doubtful about [51]. In this approach, persuasive sources can affect attitudes by affecting the number of thoughts generated. As source credibility primarily consists of expertise and trustworthiness [52], we compared an authoritative source represented by the government with a citizen-based source represented by a non-governmental organization. When the message is from trustworthy sources, it is more likely to enhance persuasion. Thus, we expect that:

**H2.**  *Information source (i.e., government vs. NGO) has an impact on the reduction in the use of disposable plastics. More specifically, the messages from the government will be more effective than those from NGOs in promoting the reduction in the use of disposable plastics.*

In addition, there is still a lack of evidence on how the combination of media frame, media modality, and information source may exert influence on behavioral intentions, so we also posed the following research question:

**RQ2:**  *How will the three-way interactions of media frame (gain vs. loss), media modality (text vs. image vs. infographic), and information source (government vs. NGO) impact the reduction in the use of disposable plastics?*

### 2.3. The Mediation Role of Emotion Responses

Previous studies found that emotions have a large influence on human behavior, in which appealing to emotions is an effective approach to persuasion [53,54]. In environmental communication, emotions may help create a greater sense of urgency for taking pro-environmental behaviors [8]. Media frames may especially evoke emotions because they foreground different degrees of situational pleasantness and outcome certainty [55]. For instance, loss frames display negative outcomes of using single-use plastics, which may evoke emotions such as fear, guilt, and sadness. In this process, perceived severity increase, and social responsibility are likely to be enhanced. Thus, negative emotions may increase participants’ willingness to engage in environmentally friendly behaviors. Gain frames present the positive outcomes of refraining from disposable plastic use, which may elicit positive emotions such as pleasure and pride. As a result, it may increase people’s efficacy and motivate pro-environmental behaviors. According to the broaden-and-build theory [56], positive emotions tend to broaden individuals’ thought-action repertoire and thus make them more open to action options. In this sense, we expect that emotion has the potential to mediate the influence of media frames on pro-environmental behaviors. Therefore, we propose the following research hypothesis:

**H3.**  *(a) Positive and (b) negative emotions will mediate the effect of media frames on the reduction in the use of disposable plastics.*

## 3. Method

### 3.1. Sample

This study used data from an online panel survey conducted in 2020 by a reputable market research company in Hong Kong. A total of 1001 Hong Kong residents were recruited from a proprietary consumer panel using gender and age quotas. The population in Hong Kong is estimated to be 7.41 million, of which 45.5% are male, with a median age of 46.3. Additionally, 82% received secondary education or above [57]. In our sample, 49.9% were male, 49.6% female, and less than 1% identified themselves as others. The median age of participants was 40 years old (*M* = 40.3, *SD* = 12.6). A total of 95.7% of participants have received secondary school education or above, and 74% had a monthly income of more than HKD 10,000. Compared with the population in Hong Kong, the sample in our study was younger and better educated.

### 3.2. Procedure

The study was conducted using an embedded experimental design that tested the effectiveness of different types of message appeals, modalities, and sources. The experimental setting was set up in the Qualtrics system, and the random assignment was conducted through Qualtrics using a randomizer tool. After providing consent, participants answered questions about their demographic information, environmental knowledge and concern, media use habit, and single-use plastics habit. Next, they were randomly assigned to receive one of our experimental stimuli.

A 2 (frame: gain vs. loss), * 3 (modality: text vs. graphic vs. infographic), and * 2 (source: government vs. NGO) between-subjects experimental design was employed in this study. Participants were randomly assigned to one of the 13 experimental conditions: group 1 (gain frame, text, and government; *n* = 74), group 2 (gain frame, text, and NGO; *n* = 75), group 3 (gain frame, graphic, and government; *n* = 78), group 4 (gain frame, graphic, and NGO; *n* = 78), group 5 (gain frame, infographic, and government; *n* = 74), group 6 (gain frame, infographic, and NGO; *n* = 79), group 7 (loss frame, text, and government; *n* = 70), group 8 (loss frame, text, and NGO; *n* = 80), group 9 (loss frame, graphic, and government; *n* = 81), group 10 (loss frame, graphic, and NGO; *n* = 79), group 11 (loss frame, infographic, and government; *n* = 77), group 12 (loss frame, infographic, and NGO; *n* = 75), and group 13 (control group; *n* = 81). Twelve versions of an ad advocating for the reduction in the use of disposable plastics were created (see Appendix A for a sample ad). The message frame was manipulated in the ad by presenting what would be gained (vs. lost) if participants refrained (vs. did not refrain) from using disposable plastics. The number of words and the themes in gain-framed and loss-framed messaging was kept the same. For instance, in the gain frame, participants read, “If we refrain from using single-use plastics, we will save the wildlife both on the land and in the oceans”; in the loss frame, they read, “If we don’t stop using single-use plastics, the wildlife both on the land and in the oceans will be devastated.” The media modality was manipulated by presenting messages with the same theme in three modalities, text, image, and infographic. The content was held as consistent as possible across conditions, albeit displayed in either text or visual format. For example, in the text (vs. image vs. infographic) condition, participants read, “If we refrain from using single-use plastics, our oceans will be saved”; in the image condition, participants are exposed to a picture of a clean ocean; and in the infographic condition, participants read the same sentence with a picture of a clean ocean. Information source was manipulated in the ad by adding a fictitious logo of a government agency vs. NGO in the message (i.e., “Environmental Conservation Department” vs. “Action for the Environment”).

After reading the stimuli, participants completed the post-test questionnaire regarding the manipulation checks and dependent measures.

### 3.3. Measures

In order to control for the factors that could affect single-use plastics reduction behaviors, participants were asked a series of demographic questions, including age, gender, education, and income level. *Age* was measured by the question, “In which year were you born?”. *Gender* was scored 1 (male), 2 (female), and 3 (Others), and *education* was assessed according to the level achieved by the respondent up to graduate degree (less than high school, high school or equivalent, college but no degree, associate degree, bachelor degree, and graduate degree). *Household income* was measured using 14 categories (ranging from 1 = under HKD 7999 to 14 = HKD 100,000 and over).

For *past behaviors*, the following 16 items were adopted to assess single-use plastics consumption frequency (see Table 1). All these items were subjected to a factor analysis using a principal components analysis with varimax rotation. The result showed that the past behaviors loaded onto a single factor with 41.7% total variance explained (*M* = 3.34, *SD* = 1.03, Cronbach’s α = 0.905).

Because single-use plastics use behavior may depend on a variety of psychological variables, we measured participants’ environmental knowledge, environmental concerns, and environmental efficacy. *Environmental knowledge* was measured by 5 items on a 7-point scale, including (1) I am very knowledgeable about environmental issues; (2) I understand the environmental phrases and symbols on product packaging; (3) I know how to buy products that are environmentally safe; (4) I know more about recycling than the average person; and (5) I know how to select products and packaging that reduce the amount of waste ending up in landfills [58]. The responses were averaged to form an additive index (*M* = 4.54, *SD* = 1.02, Cronbach’s α = 0.898). *Environmental concerns* measure individuals’ concern about the natural environment and the use of single-use plastics, including 10 items: (1) I worry about the endangered species in Hong Kong; (2) Improper disposal or dumping of industrial or domestic waste is a common phenomenon in Hong Kong; (3) The water quality in lakes, rivers, and streams is declining in Hong Kong; (4) Air pollution is a serious issue in Hong Kong; (5) It is essential to promote green living in HK; (6) I believe more environmental protection works are needed in HK; (7) Environmental protection issues are none of my business; (8) It is very important to raise environmental concerns among HK people; (9) Single-use plastics have severe negative consequences for humans and nature; (10) Recycling single-use plastics is important for the Hong Kong’s future. The items loaded onto a single factor using a principal components analysis with 49.6% of total variance explained. An additive index was created by taking the average of the responses (*M* = 5.54, *SD* = 0.809, Cronbach’s α = 0.784). *Environmental efficacy* was measured by 4 items, including (1) As long as actions are taken to reduce plastic consumption, plastic pollution can be effectively controlled, (2) I believe that I have the ability to take action to mitigate single-use plastic pollution, (3) Although it may cause inconvenience, I can still change my behavior to reduce single-use plastic consumption, and (4) I can try my best in every way to reduce plastic pollution (*M* = 3.74, *SD* = 1.05, Cronbach’s α = 0.789).

This study not only measured traditional media use and digital media use behaviors but also assessed media content consumption. More specifically, *traditional media use* was measured by three items, including watching local free-to-air TV, watching cable TV, listening to radio news, and reading traditional forms of media (e.g., newspapers, paper magazines) (*M* = 3.78, *SD* = 1.39, Cronbach’s α = 0.670). *Digital media use* was measured by four items, including the use of instant messaging apps (e.g., WhatsApp, WeChat, Snapchat), YouTube, and Facebook, and reading digitized forms of media (e.g., online newspapers, online magazines) (*M* = 4.68, *SD* = 1.37, Cronbach’s α = 0.785). *Soft news consumption* was measured by seven items, including cooking and home renovation shows, health and fitness information, movie and drama, fashion news, travel shows, celebrity news, and music videos (*M* = 4.26, *SD* = 1.07, Cronbach’s α = 0.858). *Hard news consumption* was measured by five items, including financial news, political news, environmental news, science and technology news, and nature documentaries (*M* = 4.11, *SD* = 1.10, Cronbach’s α = 0.774).

Emotional responses were measured by assessing 14 types of emotion with a 5-point scale, in which six were positive and eight were negative [59]. *The positive emotions* were feeling happy, enthusiastic, optimistic, proud, content, and excited (*M* = 1.94, *SD* = 0.953, Cronbach’s α = 0.889). *The negative emotions* include anger, fear, guilt, sadness, disappointed, nervous, worried, and frustrated (*M* = 2.46, *SD* = 0.948, Cronbach’s α = 0.928).

In terms of *the intention of refraining from using single-use plastics*, we asked respondents their willingness to engage in the following 19 behaviors, including “Bring a reusable glass or stainless steel bottle/travel mug when going out”, “Carry reusable shopping bags”, and “Avoid using plastic straws.” Answers were ranging from 1 to 7 and the responses were averaged to form an additive index (*M* = 5.06, *SD* = 0.927, Cronbach’s α = 0.923).

### 3.4. Manipulation Checks

A manipulation check was performed to ensure that the appeals were perceived as intended. The results revealed that the gain-frame message (*M* = 5.53, *SD* = 1.24) was perceived as emphasizing the benefits of not using single-use plastics compared with the loss-frame message (*M* = 5.16, *SD* = 1.47, *t* (895) = 4.18, *p* < 0.001). Meanwhile, a *t*-test indicated that respondents who were in the loss-frame message condition had significantly higher perception of loss (*M* = 5.36, *SD* = 1.33) than those who were in the gain-frame condition (*M* = 4.75, *SD* = 1.55, *t* (895) = 6.39, *p* < 0.001). Thus, both message framing conditions successfully created differential perceptions of gain and loss in participants.

In order to ensure that the stimuli were perceived as text only, graphic only, or as an infographic, participants were asked to rate their opinion of the message on a semantic differential scale that was anchored by “text-based” (1) and “visual-based” (11). As expected, the text-based message (*M* = 3.70, *SD* = 3.23) was perceived to be significantly less visual than graphic condition (*M* = 7.57, *SD* = 2.80) and infographic condition (*M* = 6.57, *SD* = 3.00).

## 4. Results

### 4.1. Effects of Experimental Stimuli

In order to examine the effectiveness of the media frame and answer RQ1, ANOVA results are displayed with means and standard deviations for all experimental groups in Table 2. The results indicated that participants who were exposed to messages with loss frames (*M* = 5.14, *SD* = 0.897) and those exposed to gain frames (*M* = 5.01, *SD* = 0.950) reported higher behavioral intentions than control group (*M* = 4.89, *SD* = 0.923; F (2, 998) = 3.91, *p* < 0.05). However, the results of post hoc show that only the effect of the loss frame was statistically significant while the gain frame was not (*p* = 0.248). Thus, only the loss frame was an effective predictor of future behavioral intention, and RQ1 was answered.

Meanwhile, compared with image-based messages (*M* = 4.96, *SD* = 0.895), text-based messages (*M* = 5.15, *SD* = 0.930) and infographic-based messages (*M* = 5.13, *SD* = 0.926) were more effective in increasing people’s intention to reduce single-use plastics use (F (2, 917) = 3.91, *p* < 0.05, see Table 3). The ANOVA results show that infographic-based message was significantly more effective than the image-based message, and thus H1b was supported and H1a was not.

Information source, however, did not show significant difference in affecting people’s behavioral intention (*M*_gov_ = 5.11, *SD*_gov_ = 0.886; *M*_NGO_ = 5.05, *SD*_NGO_ = 0.963; *t* = 1.03, *p* > 0.05, see Table 4). Therefore, H2 was not supported.

### 4.2. Moderation Effect and Mediation Effect

In order to further explore the three-way interaction effect of media frame, media modality, and information source on emotional responses and future behaviors, we conducted a MANCOVA. As shown in Table 5, after controlling for the demographic variables, psychological variables, and media use behaviors, participants exposed to text-based messages from the government were more likely to reduce their single-use plastics consumption. Moreover, loss-frame massages from NGOs were also more effective in persuading consumers to refrain from using single-use plastics. Therefore, RQ2 was answered.

In order to further explore the role of emotional responses in influencing the effect of media frames on future behaviors, this study employed a regression analysis using Model 4 in PROCESS v3.4.1 macro in SPSS. Before entering the experimental stimuli, we dummy-coded media modality and information source, which were treated as the control variables in the regression model. As shown in Table 5, negative emotion significantly mediated the relationship between gain frame and future behavioral intentions (boot 95% CI = [0.010, 0.065]), as well as between loss frame and future behavioral intentions (boot 95% CI = [0.077, 0.175]). Therefore, H3b was supported while H3a was not.

## 5. Discussion

The study examined how different presentations of the consequence of (not) refraining from using single-use plastics may affect the willingness to change behaviors, as well as the mediating role of affective arousal. The findings demonstrated that the loss frame was more effective than a gain frame in persuading individuals to reduce using disposable plastics. In addition, text and infographics were more effective than images. In particular, the study suggests a congruency between text-based messages from the government and the loss frame from NGOs conveyed by all three modalities. Moreover, the study also suggested the mediating role of negative emotion in the relationship between media frames and behavioral intentions. The study thus provided insights for both citizens and policymakers about the most effective strategies for designing messages to promote environmentally sustainable behaviors.

As Tversky and Kahneman’s study suggests, people are likely to be more accepting of risks in a loss-frame condition, whereas they are more risk-averse in a gain-frame condition [7]; later studies further discussed the contexts in which the gain and loss frames work. This study provides new evidence that for pro-environmental behaviors such as refraining from using disposable plastics, the loss frame is more persuasive than the gain frame and is more likely to foster intentions to avoid undesirable behaviors. The finding is consistent with studies conducted in the Western contexts [60,61,62]. This can be explained that negative information is often perceived as more important and salient, which may induce emotions such as fear and guilt and trigger further behavior change [63].

Interestingly, compared with images, text and infographics are more effective in persuading people to reduce using disposable plastics. One possible reason is that text and infographics provide more detailed information and require more time to process the information, which may stimulate cognitive activity and motivate the central route of information processing [44]. Graphical representation, in contrast, seems to constrain information elaboration and lead to a peripheral route of processing as ELM predicts [47].

The government source, in general, does not show a significant difference from the NGO source in affecting participants’ behavioral intentions. As pointed out by some scholars, the environmental governance in Hong Kong relies on both the governmental authorities and social actors, and in particular, social actors are often the first movers in environment-related policies [64]. This finding could be specific to Hong Kong as, in recent years, the government has faced a crisis of legitimacy due to the absence of universal suffrage and the imposition of the National Security Law, resulting in a low political trust in the government among Hong Kong citizens [65]. Furthermore, Hong Kong citizens also distrust the recycling industry, with many calling for the recycling industry to be more transparent and be held accountable for its practices. Such calls are aimed at bolstering the actualization of Hong Kong’s “waste reduction, resources circulation, zero landfill” [66], as stated in the Hong Kong government’s Waste Blueprint for 2035. The findings also suggest the moderating effect of information sources on media modality and media frame. In detail, the effect of text is more prominent when it is from the government source, and the effect of the loss frame will be strengthened when it is from the NGO source. According to the construal level theory (CLT), an individual’s level of abstraction systematically influences cognitive processing, and abstraction often makes mental representations more inclusive and focused on central characteristics [67]. Different from images focusing on concrete and low-level details, written texts are more abstract and with greater attention to higher-level concepts. When exposed to text-based messages, people are more likely to activate high-level construals and focus on the importance (i.e., gain and loss) of engaging in certain behaviors and paying less attention to the information source. This may provide an explanation for why messages from the government are more persuasive when they are conveyed by text, with either the gain or loss frames. The loss frame, associated with a higher level of negative emotions, tends to be more effective when released by NGOs among all three modalities. One explanation is that in Hong Kong, people are more likely to trust environmental NGOs when considering the negative consequences of certain behaviors, especially when associated with taking risks and making sacrifices. As NGOs usually keep a distance from the center of political power, the loss-frame messages from them are perceived as more realistic for ordinary citizens, thus leading to more favorable outcomes.

This study also demonstrates the mediating role of negative emotions. Compared with positive emotions, negative emotions such as fear, anxiety, and guilt are often linked to higher arousal, which may lead to motivated avoidance behavior [68]. For instance, emotions such as guilty have the potential to enhance perceptions of social responsibility, which motivate people to confess and amend behaviors [8]. In order to reduce negative emotions, people are more willing to take action and act in a socially and environmentally responsible way. This finding is in line with previous studies on sustainable behaviors [19,69]. In addition, the role of negative emotions was widely discussed by the Protection Motivation Theory [70] and the Health Action Process Approach [71] in health domains, which describes how an individual copes with stressful situations and highlights the influence of fear emotion on the persuasion process.

There are several limitations of this study that should be considered when interpreting the results. First, this study focused on the gain vs. loss framing based on the prospect theory, while there are other ways of framing that may also play an important role in curbing plastic consumption. For instance, Septianto and Lee demonstrated different effects of a “why” message (e.g., reasons to reduce plastic consumption) vs. a “how” message (e.g., steps to reduce plastic consumption) [72]. Future studies may explore other possibilities of media framing to encourage recycling behaviors. Second, as we were interested in the effects of media modalities on sustainable behaviors, messages were designed in text, images, and infographics. Although we kept the content as consistent as possible across the three modalities, it is still likely that images may present less information than infographics. Third, as mentioned earlier, the gain and loss frames emphasize the benefits of refraining from using single-use plastics or the costs of not performing this behavior, which may contain an implicit efficacy induction.

Finally, since this study was conducted among Hong Kong residents only, future research should aim to compare the effectiveness of the above campaign strategies in curbing the use of disposable plastics in other political and cultural contexts. Future studies should also explore different ways of segmenting target audiences so that effective intervention strategies can be designed with specific groups in mind.

## 6. Conclusions

In this study we examined how the effectiveness of public engagement campaigns aimed at reducing the use of disposable plastics can be optimized by experimentally testing the effects of message frame, modality, and information source on behavioral intentions. The findings show that the loss frame was more effective than the gain frame in reducing the future use of disposable plastics. We also demonstrate that, compared to image-based messages, text-based and infographic-based messages were more effective in promoting the reduction in the use of disposable plastics, but find no main effect of information source. Finally, our study highlights an important mediating role of negative emotions in promoting environmentally sustainable behaviors.

## Figures and Tables

**Table 1:** Factor analysis of single-use plastics behaviors.

	Factor Loading	Cronbach’s α
Use plastic stirrers delivered in your drinks	0.736	0.905
Use plastic cups and utensils at social events	0.731	
Purchase disposable plastic bottles of water	0.708	
Use plastic straws delivered in your drinks	0.685	
Use plastic tape to wrap gifts or for other types of packaging needs	0.681	
Purchase sodas, juices, and other plastic-bottled beverages	0.663	
Use plastics bags offered at malls to hold your wet umbrella	0.661	
Pay for plastic bags offered at stores	0.651	
Purchase food in disposable cups, plates, and cutlery in the lunchroom	0.641	
Use disposable utensils and cups in food delivery	0.635	
Purchase foods or fruits that are plastic-packaged in the supermarket	0.619	
Use disposable plastic pens	0.589	
Use plastic freezer bags to preserve foods	0.582	
Use single-use plastic gloves	0.580	
Use disposable plastic hair brush, comb, toothbrush, or shower cap offered by hotel	0.572	
Use cotton buds in daily life	0.560	

**Table 2:** ANOVA results for behavioral intention with means and standard deviations for message frames.

	N	M	SD	Sig.
Frames				
Gain frame	458	5.01	0.950	
Loss frame	462	5.14	0.897	
Control	81	4.89	0.923	
Mean difference between control group vs. gain-framing group				
		−0.129	0.111	0.248
Mean difference between control group vs. loss-framing group				
		−0.258	0.111	0.020

F (2, 998) = 3.91, *p* < 0.05.

**Table 3:** ANOVA results for media modality.

	N	M	SD	Sig.
Modality				
Text	299	5.15	0.930	
Image	316	4.96	0.895	
Infographic	305	5.13	0.926	
Mean difference between text-based group vs. infographic-based group				
		0.021	0.075	0.779
Mean difference between image-based group vs. infographic-based group				
		−0.168	0.074	0.024

F (2, 917) = 3.91, *p* < 0.05.

**Table 4:** Independent *t*-test for information source.

	N	M	SD	*t*	Sig.
Information Source					
Government	454	5.11	0.886	1.03	0.303
NGO	466	5.05	0.963		

**Table 5:** Predictors of emotional responses and future behavioral intention.

	Positive Emotion	Negative Emotion	Future Behavior
	B (SE)	B (SE)	B (SE)
*Control variables*			
Age	0.002 (0.003)	−0.001 (0.002)	0.010 (0.002) ***
Gender (male = 1)	−0.115 (0.062)	−0.091 (0.060)	0.164 (0.060) **
Education	0.055 ** (0.021)	0.022 (0.021)	0.022 (0.021)
Income	−0.031 *** (0.009)	−0.001 (0.009)	0.028 (0.009) **
*Psychological variables*			
Env. concern	−0.377 *** (0.039)	0.035 (0.040)	0.440 (0.034) ***
Env. knowledge	0.286 *** (0.031)	0.116 *** (0.031)	0.164 (0.026) ***
Env. efficacy	0.095 *** (0.029)	0.068 * (0.030)	0.159 (0.025) ***
*Media use behaviors*			
Traditional media	0.121 *** (0.025)	0.090 *** (0.026)	0.015 (0.022)
Digital media	−0.036 (0.024)	0.010 (0.025)	0.042 (0.021) *
Soft news	0.096 * (0.038)	0.070 (0.039)	−0.003 (0.033)
Hard news	0.050 (0.038)	0.048 (0.039)	0.139 (0.033) ***
*Main effects of experimental stimuli*			
Frame (gain frame = 1)			
Loss frame	−0.291 *** (0.055)	0.580 *** (0.055)	0.116 * (0.047)
Modality (image = 1)			
Text	0.175 * (0.068)	0.240 *** (0.070)	0.113 (0.060)
Infographic	0.241 *** (0.068)	0.196 ** (0.070)	0.091 (0.059)
Information Source (NGO = 1)			
Government	0.020 (0.056)	0.111 (0.059)	0.027 (0.049)
*Interactions* (control = 1)			
Gain * text * gov.	−0.011 (0.135)	0.834 *** (0.136)	0.289 * (0.119)
Loss * text * gov.	−0.127 (0.137)	1.20 *** (0.138)	0.288 * (0.121)
Gain * text * NGO	−0.064 (0.135)	0.951 *** (0.136)	0.109 (0.119)
Loss * text * NGO	−0.197 (0.133)	1.28 *** (0.134)	0.290 * (0.117)
Gain * image * gov	−0.141 (0.134)	0.620 *** (0.135)	0.123 (0.118)
Loss * image * gov	−0.469 *** (0.133)	1.42 *** (0.134)	0.177 (0.117)
Gain * image * NGO	−0.002 (0.134)	0.719 *** (0.135)	−0.017 (0.118)
Loss * image * NGO	−0.483 *** (0.133)	1.36 *** (0.134)	0.245 * (0.117)
Gain * infog. *gov	0.153 (0.135)	0.936 *** (0.136)	0.205 (0.119)
Loss * infog. *gov	−0.137 (0.134)	1.09 *** (0.135)	0.208 (0.118)
Gain * infog. * NGO	0.106 (0.133)	0.803 *** (0.134)	0.203 (0.117)
Loss * infog. * NGO	−0.264 * (0.135)	1.26 *** (0.136)	0.263 * (0.119)
*Emotional responses*			
Positive emotion			−0.024 (0.029)
Negative emotion			0.077 ** (0.029)
*Indirect effect tests* (*message frame* via *emotional response on future behaviors*)			
	Effect (BootSE)	LLCI	ULCI
Gain frame → Positive emotion → future behaviors	0.008 (0.008)	−0.009	0.024
Loss frame → Positive emotion → future behaviors	−0.004 (0.006)	−0.017	0.006
Gain frame → Negative emotion → future behaviors	0.039 (0.014)	0.010	0.065
Loss frame → Negative emotion → future behaviors	−0.064 (0.026)	−0.119	−0.018
Total	0.123 (0.025)	0.077	0.175

Note. *** *p* < 0.001, ** *p* < 0.01, * *p* < 0.05.

**Figure A1:**
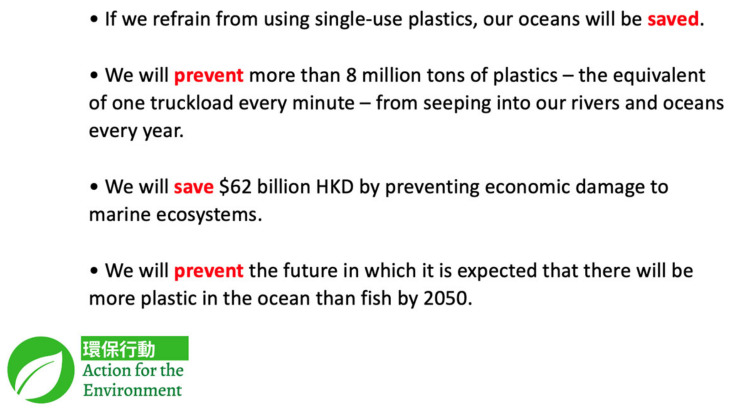
Example of condition 1: gain * text * NGO.

**Figure A2:**
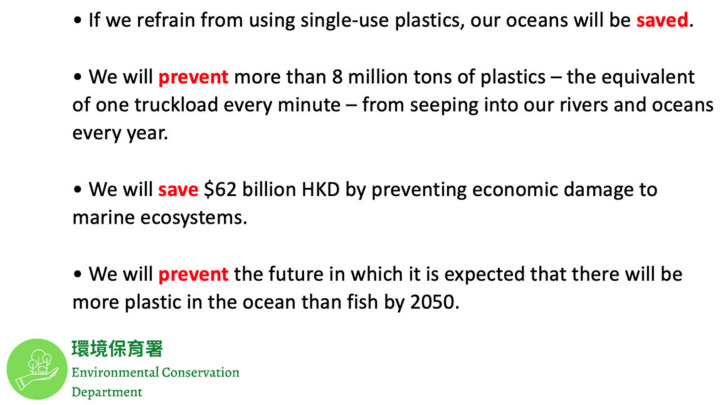
Example of condition 2: gain * text * government.

**Figure A3:**
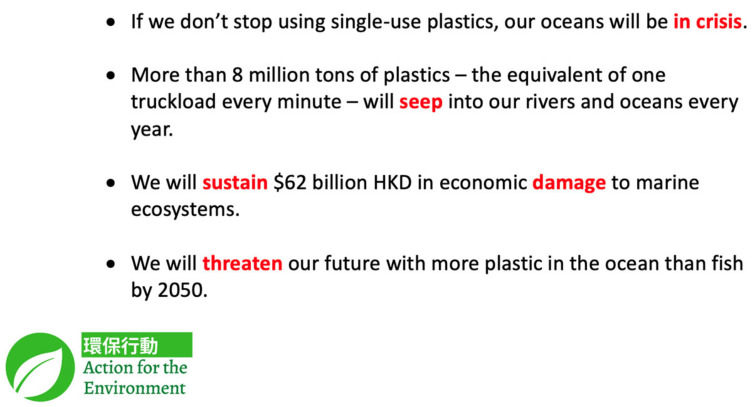
Example of condition 3: loss * text * NGO.

**Figure A4:**
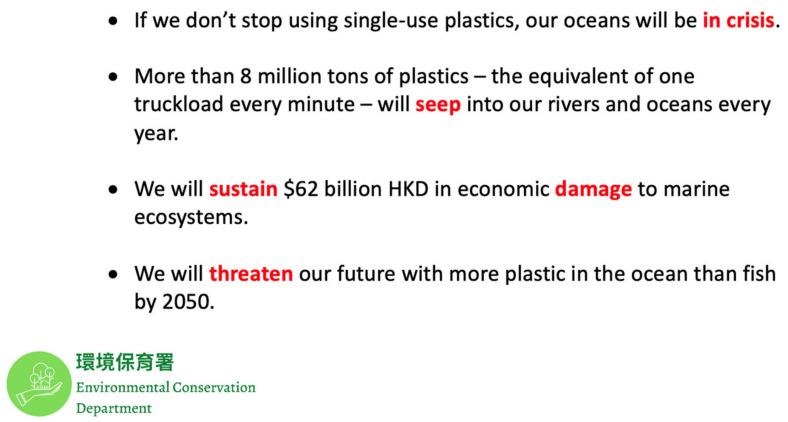
Example of condition 4: loss * text * gov.

**Figure A5:**
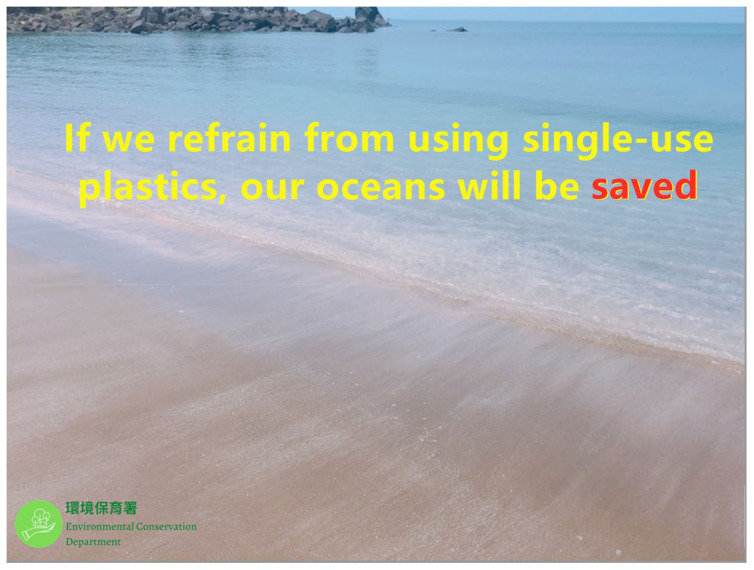
Example of condition 5: Gain * image * government.

**Figure A6:**
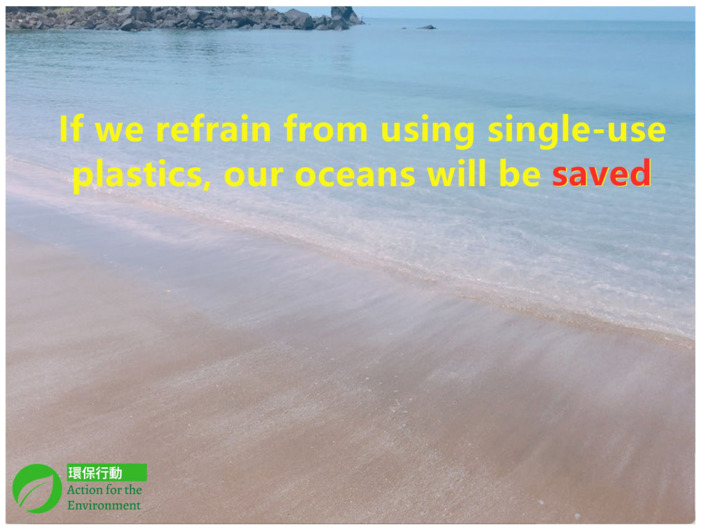
Example of condition 6: gain * image * NGO.

**Figure A7:**
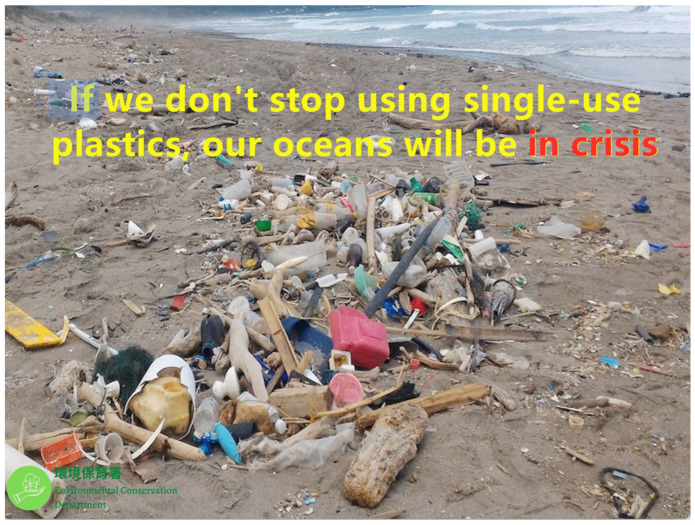
Example of condition 7: Loss * image * government.

**Figure A8:**
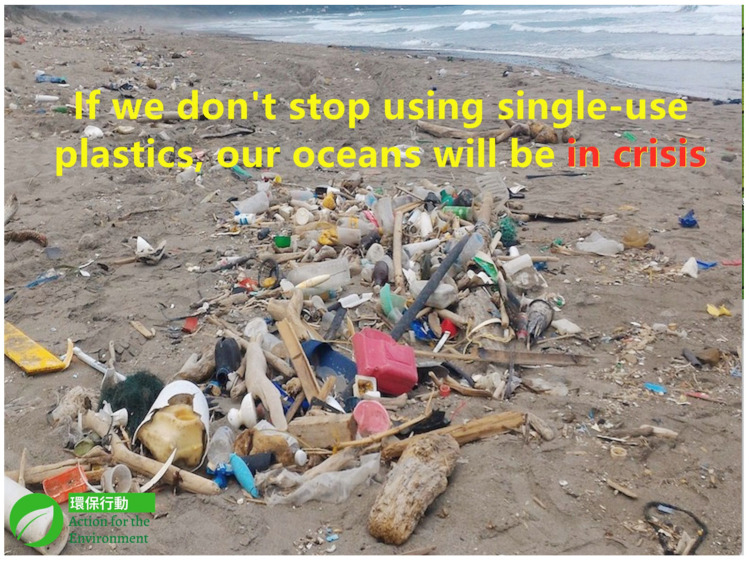
Example of condition 8: Loss * image * NGO.

**Figure A9:**
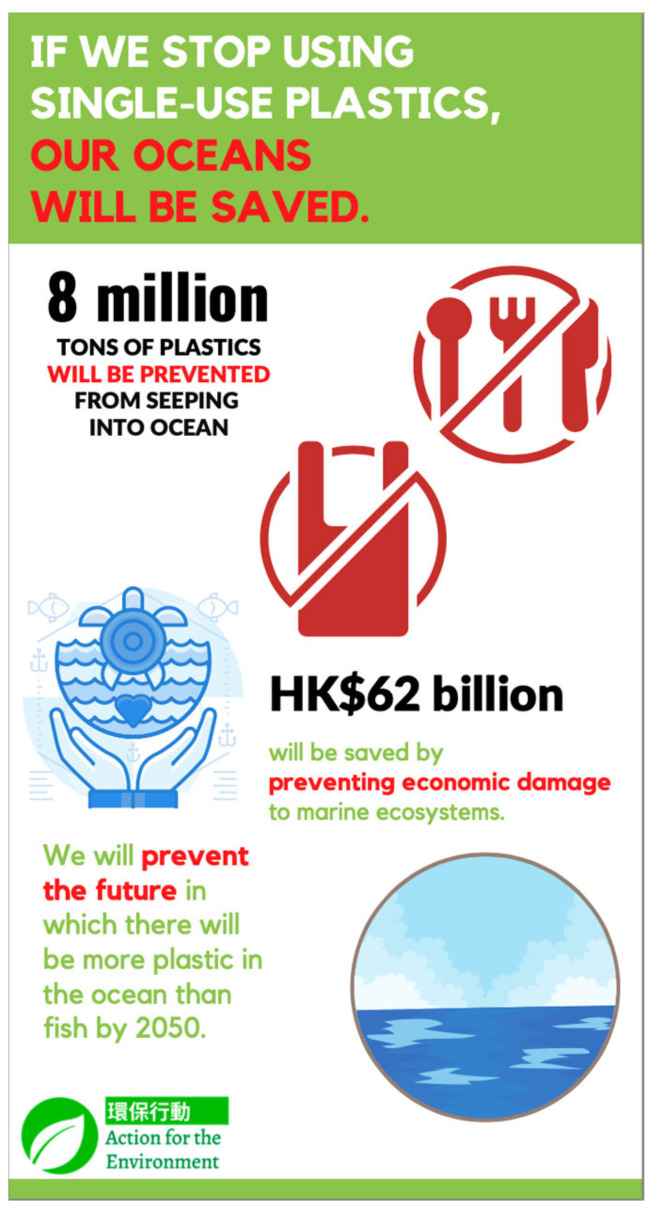
Example of condition 9: gain * infographic * NGO.

**Figure A10:**
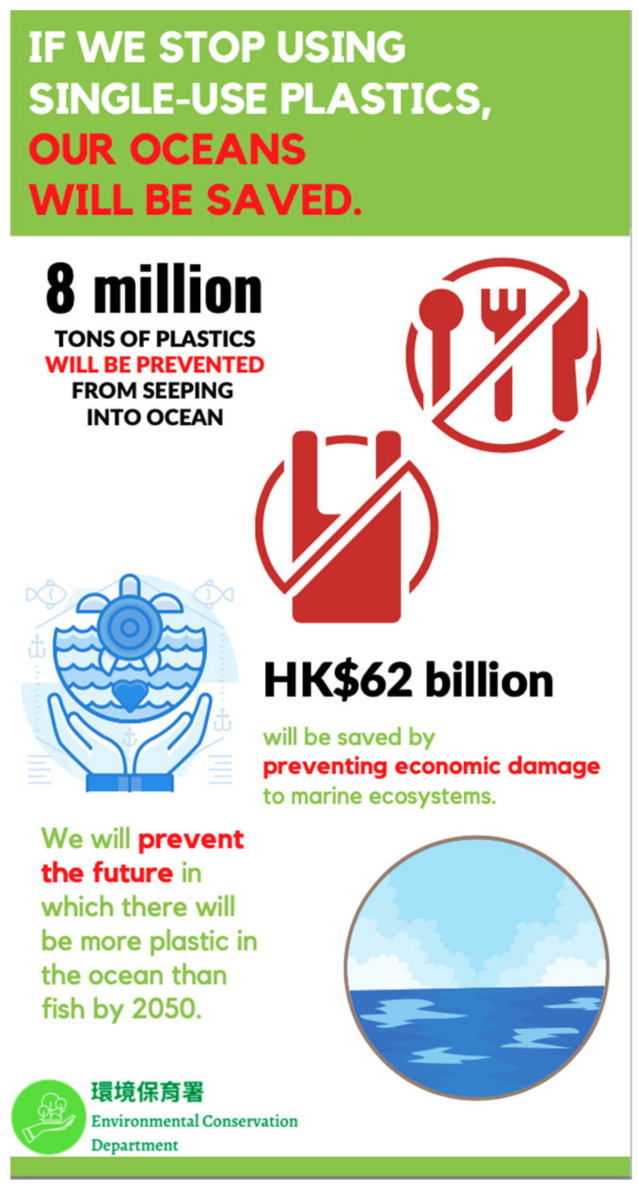
Example of condition 10: gain * infographic * government.

**Figure A11:**
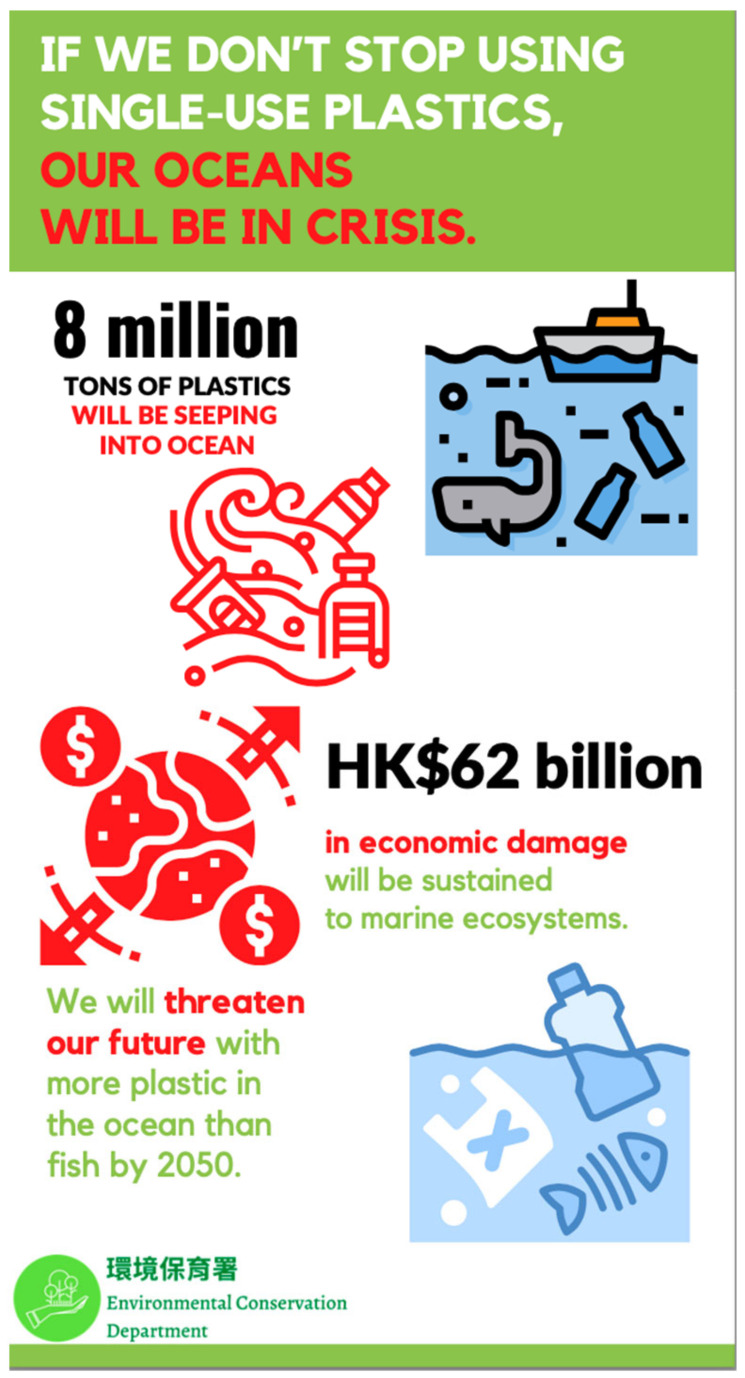
Example of condition 11: loss * infographic * government.

**Figure A12:**
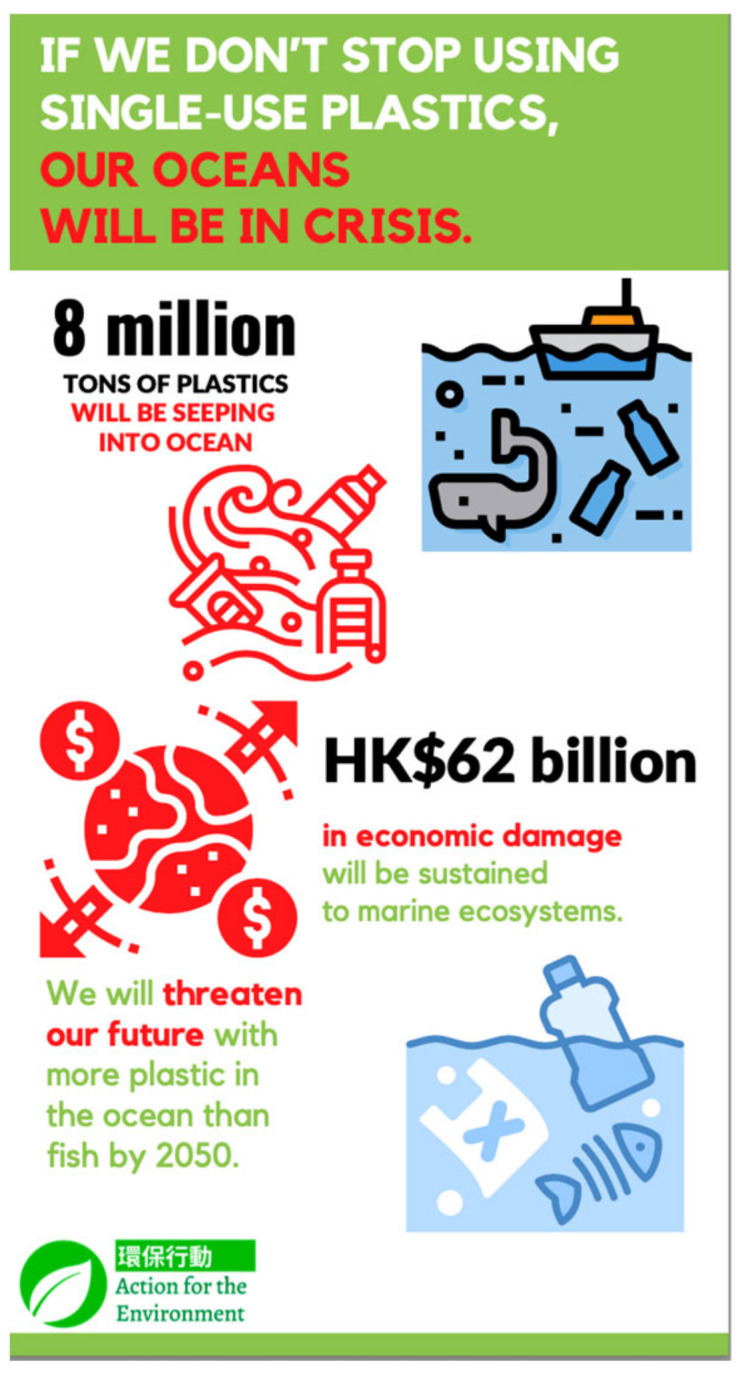
Example of condition 12: loss * infographic * NGO.

**Figure A13:**
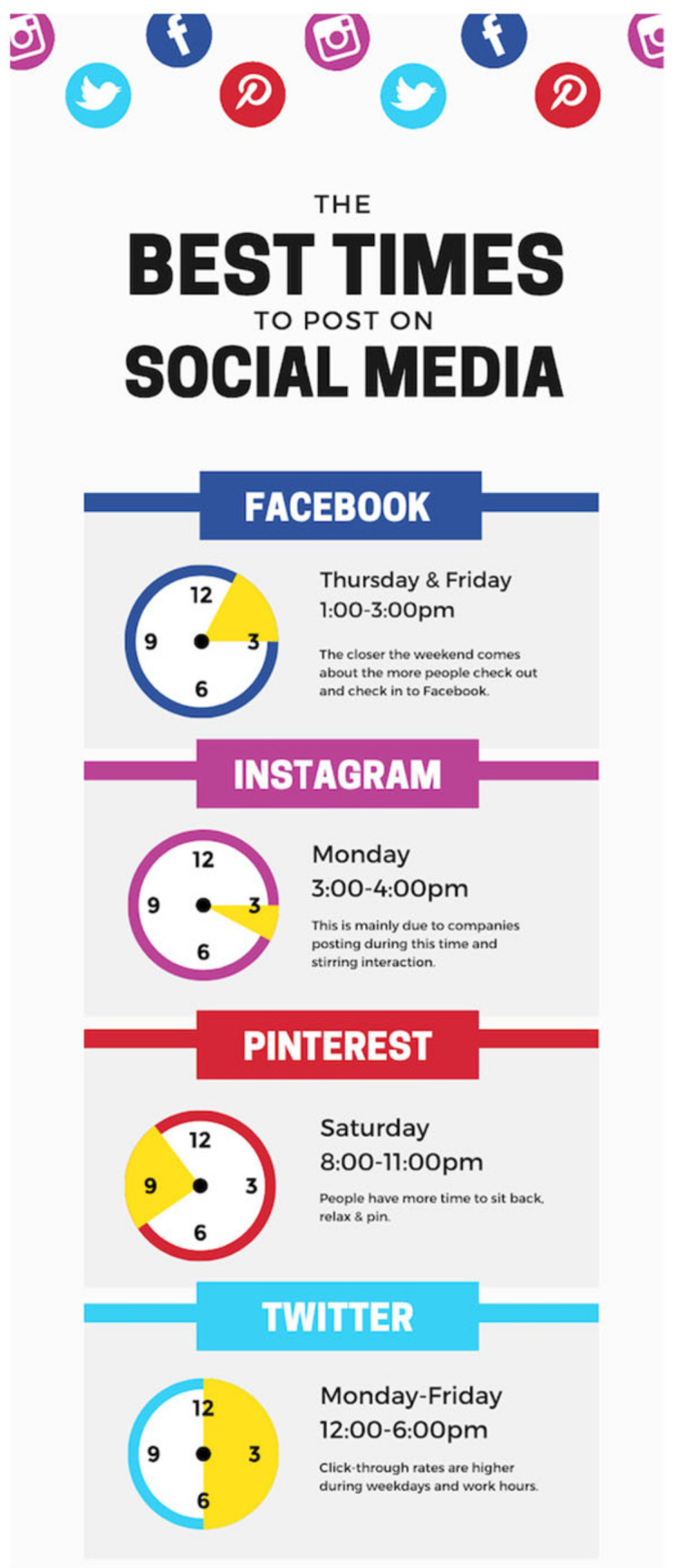
Example of condition 13: control group.

## Data Availability

Data from this study are available upon request.
